# Cornered professors, a fragilized model, and graduate training… who holds it together?

**DOI:** 10.17179/excli2026-9340

**Published:** 2026-03-11

**Authors:** Mairim R. Serafini, Lucindo J. Quintans-Júnior

**Affiliations:** 1 Graduate Program of Health Science (PPGCS), Federal University of Sergipe (UFS), Aracaju, Sergipe, Brazil

## ⁯⁯⁯

Few relationships in academic life are as intense, prolonged, and emotionally charged as the one between advisor and advisee in graduate education. It is an asymmetrical bond by definition, permeated by expectations, frustrations, idealizations, disappointments, and symbolic disputes. It is unique because people do not repeat themselves, even when they occupy the same institutional space. Yet public debate insists on treating this relationship in a simplistic manner. On one side, the student is elevated to the status of a permanently vulnerable subject. On the other, the professor is reduced to an automatically suspect figure. This shallow reading does not clarify the problem; it merely impoverishes it and makes both parties ill.

It is evident that abusive practices exist in academia. There is authoritarianism, humiliation, moral harassment, and the perverse use of power, and these must be named and confronted (Björklund and Jensen, 2025[2]). This debate is necessary and deserves focused attention, especially at a time when the growing number of students and faculty taking leave due to depression and other mental health conditions signals that something deeper is collapsing. The blind spot, however, emerges when the standard shifts and every demand is interpreted as violence, every boundary as oppression. In this confusion, something fundamental is lost: the distinction between abuse and rigor, between authoritarianism and formative responsibility.

Psychology and psychoanalysis help explain, at least in part, why this confusion has become so frequent. Freud already noted that any relationship with authority figures reactivates childhood contents: fear of making mistakes, the need for approval, excessive sensitivity to criticism (Schlosser et al., 2003[4]). When an advisor cuts a paragraph, denies a premature advance, suggests a safer path learned through experience, dares to propose unexplored shortcuts, or simply points out a methodological flaw, they directly confront the student's narcissistic investment - that is, the image the student has built of themselves as competent or already “ready.” Graduate education, however, is precisely the opposite: a formative process.

Under high emotional load, criticism is no longer processed as corrective information but perceived as a personal threat. Attention shifts from the work itself to the self, triggering defensive responses that preserve subjective integrity at the expense of learning. As Lacan emphasized, learning requires accepting not-knowing; thus, the advisor's role is structural, not personal, in sustaining the boundary between scientific rigor and the illusion of mastery. However, this function is increasingly misread as authoritarianism. In a context where empathy is equated with the absence of frustration, confrontation with error, limits, and complexity is avoided, impoverishing academic formation. From a Jungian perspective, such conflicts often reflect projected “shadow” contents - envy, resentment, rivalry, or inadequacy - where the advisor becomes the locus of discomfort rather than its cause.

Little attention is paid to the other pole of the advising relationship: situations in which students repeatedly cross boundaries through disrespect, affective manipulation, or disregard for agreed norms, turning advising into a persistent field of conflict. In such contexts, teaching becomes marked by excessive caution and strategic silence, undermining dialogue and contributing to faculty distress. This suffering, however, is often silenced or naturalized. As a result, a gap persists in the literature, with few studies addressing harassment directed at faculty members. Although less visible and less frequently acknowledged than student-focused harassment, these pressures are equally damaging, capable of compromising academic trajectories and eroding careers, yet remain largely neglected due to their diffuse and normalized nature.

A recent Brazilian survey reveals an alarming figure: eight out of ten teachers have considered leaving the profession. Although specific data for higher education are lacking, this malaise runs through universities (Tokarnia, 2024[5]). There is growing discouragement toward teaching, researching, and advising in graduate programs - not because of science itself, but due to environments perceived as hostile, marked by constant surveillance, diffuse judicialization, and bureaucracy that falls almost exclusively on those in the middle of the process. The effect is predictable: continuous states of alert, exhaustion, and withdrawal. We lose good faculty under the weight of silent harassment, the sense of the futility of effort, and chronic overload. In this context, the question ceases to be why so many give up and becomes another, much simpler one: why continue?

There is also a structural point that is rarely discussed: the need to recognize different roles in the academic training process. This has nothing to do with authoritarianism or abuse of power, but with the very logic of teaching and research. The advisor occupies this position not because they are “superior,” but because they have more experience, are institutionally responsible for the work, and represent the criteria, deadlines, and demands of science. For example, the expansion of predatory journals represents a real risk (Quintans-Júnior et al., 2023[3]); the advisor's experience is essential to avoid these pitfalls and to direct scientific output toward reputable and well-recognized journals.

When this asymmetry is denied, equality is not produced, but confusion. The student places themselves on the same decision-making level without having yet gone through the necessary formative path (Babich, 2023[1]). The advisor, in turn, is prevented from exercising their function. The result is not emancipation, but the disorganization of the bond, the impoverishment of training, and increasingly unpromising outcomes for research and for those who should be transformed by the process: the advisees.

It is necessary to state something that seems to have become unsayable: not all discomfort is violence. Not all demands are abuse. Not all pain is injustice. Graduate education is not a therapeutic space, although it does summon deep affects. It is, above all, a space of intellectual and ethical formation, which requires tolerance for frustration, subjective responsibility, and the capacity to listen. By infantilizing the student, we deprive them of the chance to mature. By demonizing rigor, we empty the very idea of academic training. And by silencing the suffering of advisors, we create an environment in which limits become taboo and conflict becomes pathology (Babich, 2023[1]).

Perhaps it is time to broaden the question. Instead of discussing only who protects the student, we must also ask: who supports the advisor? Who legitimizes the responsible exercise of limits? Who recognizes that scientific training requires tension, confrontation, and time?

Thus, this dynamic has particularly corrosive effects on the training of health professionals, especially physicians, whose education necessarily depends on exposure to uncertainty, error, and rigorous critical scrutiny. The systematic victimization of the young researcher discourages engagement with the healthy and unavoidable stress inherent to respectful confrontation of ideas, hypotheses, and clinical reasoning. Instead of learning to tolerate disagreement, revision, and limits - core elements of professional maturation in medicine - students are incentivized to avoid conflict and intellectual tension. The result is a weakened formative process, marked by reduced critical autonomy, lower resilience, and diminished capacity to assume responsibility in complex decision-making contexts that characterize professional practice in health care.

Until this conversation is held honestly, we will continue to call growth violence, critique attack, and rigor abuse. The outcome is predictable. In the name of a poorly understood form of protection, the university gradually relinquishes precisely what it should preserve: the formation of intellectually solid, emotionally responsible subjects, capable of sustaining - without outsourcing - their own desire to know.

## Notes

Mairim R. Serafini and Lucindo J. Quintans-Júnior (Graduate Program of Health Science (PPGCS), Universidade Federal de Sergipe (UFS), Aracaju, Sergipe, Zip code: 49060-108, Brazil; E-mail: lucindo@academico.ufs.br) contributed equally as corresponding author.

## Declaration

### Conflict of interest

The authors declare no conflict of interest.

### Artificial Intelligence (AI) - assisted technology

An AI tool was used solely for English language correction and writing refinement, without any contribution to the creative or scientific content of the manuscript.

### Acknowledgments

The article was originally conceived and written by Prof. Mairim R. Serafini, who kindly invited me to collaborate on the development of the manuscript. So, I am grateful for the opportunity to contribute to such a highly relevant topic, particularly given its recurrent neglect in contemporary academic debate.
